# Health Professionals' Knowledge and Attitudes About Neonatal Bathing and Factors Affecting Them: A Cross-Sectional Study

**DOI:** 10.1155/jonm/9970368

**Published:** 2025-04-10

**Authors:** Aysegul Simsek, Serap Özdemir, Suzi Özdemir

**Affiliations:** ^1^Department of Pediatric Nursing, Faculty of Health Sciences, Marmara University, Istanbul, Turkey; ^2^Department of Pediatric Nursing, Faculty of Health Sciences, Gaziantep University, Gaziantep, Turkey; ^3^Department of Midwifery, Faculty of Health Sciences, Kocaeli University, Kocaeli, Turkey

**Keywords:** bathroom, health professional, newborn, nurse

## Abstract

In pediatrics, the knowledge and approach of health professionals in bathing, which are one of the first care of the newborn, are important in the initiation and continuation of care by transferring it to parents. The aim of the study was to determine the knowledge, attitudes, and influencing factors of health professionals about newborn bathing. This descriptive and cross-sectional study was conducted with 140 health professionals working in neonatology between March and June 2023. Data were collected and analyzed using the “Participant description form” and “Knowledge and Attitudes Form on Newborn Bathing.” Since there is no equivalent measurement tool in the literature, the Knowledge and Attitude Form on Newborn Bathing was designed and submitted for expert assessment. A final version with 21 items questioning knowledge (15 items) and attitude (6 items) was completed. The cutoff point in the form was determined as the correct response of 16 items (12 items or more for the knowledge and 4 items or more for the attitude section). Descriptive and comparative analyses were performed on the data. Significance was evaluated at 95% confidence interval. A total of 140 health professionals with a mean age of 31.4 years participated in the study. The first bath of the newborn was reported to be a wipe/sponge bath with 80% and 68.6% reported that the first bath should be with water only. About 70% reported that there were clues indicating that the newborn was ready for bathing and the first clues were absorption of vernix caseosa (22.6%) and umbilical cord shedding (11.9%). According to the answers of the Knowledge and Attitudes Form on Newborn Bathing, 74.3% of the participants had sufficient knowledge about bathing. A statistically significant difference was found between gender, occupation, education, institution, clinic, working time, and knowledge adequacy (*p* < 0.05). Although bathing seems harmless, it is ill-advised when it comes to newborns. The study determined that health professionals possess sufficient knowledge about newborn bathing. We recommend studies be conducted on how health professionals transfer their knowledge to mothers with new babies and how mothers apply their instructions and advice.

## 1. Introduction

Skin care is one of the most important care practices in the adaptation of the newborn to the outside world after birth and should be performed without disrupting the barrier functions of the skin and without harming the newborn [1]. After birth, the newborn skin uses the vernix caseosa to adapt to infection, radiation, heat imbalances, and loss [2]. Newborn bathing is one of the first skin care practices applied for the first time in the child's life. Bathing practices are therefore important in skin protection and homeostasis [3]. Proper care of the stratum corneum prevents transepidermal fluid losses and maintains heat balance [4]. Bathing is an application that is economical, protects the skin, increases family satisfaction, and provides comfort to the newborn [4, 5]. Bathing eliminates skin contamination in the hospital environment and contamination from family and baby interaction and touching [2, 6–8]. It has hygienic, esthetic, and cultural and individual benefits [1, 9].

The literature provides little knowledge about bathing methods, properties of appropriate cleaning agents, water temperature, or bathing time. In the first days of life, newborns can be given a wipe bath or a full bath (bathtub). In recent years, cleaning products containing only warm water or soap are recommended for the first bath [10]. The National Association of Neonatal Nurses (NANN) recommends warm water or a moisturizing, neutral pH cleanser to remove amniotic fluid and, if necessary, blood [11]. The UK does not recommend the addition of lotions or cleansing products to bath water, and states that a mild, unscented soap can be used [12]. Newborn bath types are classified as wiping/sponging, tub immersion, wrapping, and shower. Tub bathing may also include immersion bathing without washing the head [12, 13]. Recently, swaddling baths have also been recommended for newborns [14, 15]. It has been reported in the literature that bathing should be at least 2-3 times a week and the duration of bathing should not exceed 5–10 min [16, 17]. Baths less than 5 min in duration can prevent skin irritation [12].

Inappropriate bathing (premature bathing, complete removal of varnish from the skin, temperature imbalance, etc.) may bring some risks (disruption of the skin barrier, hypothermia, stress). Bathing is therapeutic, considering variables such as temperature and duration. The baby both copes with stress, and the strength of the skin barrier may increase. Bathing is a stressful event for the newborn as well as for the parents who give the bath [8, 18]. In a study conducted with 288 mothers in the postpartum period, it was determined that mothers wanted to receive knowledge about baby bathing [19]. Parents should be informed about bathing according to the sociocultural environment in which they live. For example, the World Health Organization (WHO) recommends delaying the first bath for 24 h unless there is a medical or cultural issue [20]. This is why parents need adequate and accurate information about baby bathing.

For these reasons, it is important for health professionals to inform parents about this issue in bathing practices. Determining the knowledge, attitudes, and skills of neonatal healthcare professionals about newborn bathing and developing and implementing a common accepted protocol for newborn bathing further the care of babies in the neonatal unit and the wellbeing of the family after discharge. Therefore, health professionals caring for newborns are expected to have basic knowledge and skills about newborn bathing. In addition, they should follow current information and apply it in the units where they work and recommend it in parent education. This study evaluates the knowledge, attitudes, and influencing factors of health professionals working in the field of neonatology about newborn bathing.

### 1.1. Research Questions

1. Is the level of knowledge and attitudes of health professionals about neonatal bathing adequate?
2. Is there a difference in knowledge and attitudes about newborn bathing among health professionals?
3. Do the individual characteristics of health professionals affect the knowledge and attitudes about neonatal bathing?

## 2. Materials and Methods

### 2.1. Design and Type of Research

The research was designed as cross-sectional and descriptive type.

### 2.2. Study Participants

The study population consists of health professionals working in the field of pediatrics who could be reached between January 2023 and June 2023. No sample selection was made, and the study was completed with those who met the inclusion criteria and agreed to participate in the study. The health professionals participating in the study work in hospitals in Istanbul (10 public and 3 private hospitals). The health professionals in the study consisted of people working in different professional groups (physicians, nurses, midwives) and in the field of pediatrics in many hospitals. The study was completed 140 health professionals who met the inclusion criteria, working in a neonatal intensive care unit (NICU), pediatric clinics, or delivery room; qualification as a health professional (midwife, physician, and nurse); at least a bachelor's degree and agreeing to participate in the study.

### 2.3. Data Collection Tools

Based on examples in the literature, the researchers prepared a Participant description form and the “Knowledge and Attitudes Form on Newborn Bathing.”

The Participant description form collected demographic information such as the participant's age and gender and professional information such as field of expertise, educational status, and working hours.

Since no similar assessment tool was found in the literature, the “Knowledge and Attitude Form on Newborn Bathing” was prepared. The form consists of 21 items. Fifteen items question knowledge (5, 7, 8, 9, 10, 10, 11, 12, 13, 14, 15, 17, 18, 19, 19, 20, 21), and 6 items question attitude (1, 2, 3, 4, 6, 16). Statements are answered as true and false. True statements were scored as 1 and false 0 (Table 1). After the items were created for the form, language and scope appropriateness were questioned. The form was sent to 10 experts (physicians, nurses, and midwives) and their opinions were obtained. Language and content validity were made in the expert opinion. The form was edited in line with their suggestions. Then, a pilot study was conducted in terms of comprehensibility. The pilot study consisted of 10 people who met the inclusion criteria. Then, the final version of the information form was completed. Those who participated in the pilot study were not included in the main study. The total item cutoff point of the form was determined as the correct response of 16 items. In the attitude section, 4 answers and above in the attitude section and 12 answers and above in the knowledge section indicate that the level of knowledge is sufficient. The form is a test that evaluates knowledge and attitudes, and an evaluation was made based on the cutoff point.

### 2.4. Data Collection

After obtaining the necessary ethics committee permission, the data collection phase started. The study was conducted in an online platform. The link to the data collection tools was sent to the participants via Internet-based messaging applications. In the transferred form, the participants were first informed about the study, and the button to approve the study appeared. The questions on the form were displayed to the participants who selected the option “I approve to participate in the study” and they were asked to answer them. The link to the form was sent to the first health professional (a nurse) and asked to send it to their acquaintances. The average response time for the online questionnaire was 10–15 min.

### 2.5. Ethical Dimensions of Research

Before the study started, written permission was obtained from the Human Research Ethics Committee of Istinye University, Istanbul, Turkey (Approval number: 2023/3). The Helsinki Declaration of Human Rights was complied with in the study. The consent form was presented to participants online; after participants gave their electronic consent, the survey questions were released.

### 2.6. Data Analysis

The Knowledge and Attitudes Form on Newborn Bathing, prepared based on examples in the literature, was submitted for evaluation by experts before the study began. The content validity index was calculated. ROC analysis based on correct answers determined 16 correct answers as the benchmark of minimum sufficient knowledge.

Data from the study were analyzed with a computer-aided statistical program (SPSS, v26). In the analysis of numerical data, mean, median, standard deviation, and minimum and maximum values were calculated; in the analysis of categorical data, number and percentage values were calculated. Normality distributions of the data were made using Mann–Whitney *U*-test and Kruskal–Wallis tests. Bonferroni and Tamhane post hoc tests were used to determine the groups originating significance. Significance will be accepted as *p* < 0.05 within the 95% confidence interval.

## 3. Results

The study was completed with 140 participants of average age 31.4 years.  Table 2 presents demographic characteristics of the participants, and Table 3 presents their thoughts about newborn bathing.

Table 1 shows the distribution of participants' knowledge and attitudes about newborn bathing.  Table 4 presents health professionals' answers to the questions in the Knowledge and Attitudes Form on Newborn Bathing and compares the newborn bath knowledge and demographic characteristics of health professionals. When the cutoff point of the items in the form was analyzed, giving the correct answer to 16 or more statements was found to be sufficient; 74.3% (*n* = 104) of respondents answered 16 or more items correctly. A statistically significant difference was found between gender, profession (midwife, physician, nurse), working unit, institution, total and last working unit working time and education level, and newborn bath knowledge (*p* < 0.005).

## 4. Discussion

Many studies in the literature examine the knowledge levels of health professionals regarding the basic care of the newborn and newborn skin care [20–22]. The newborn bath affects the thermoregulation of newborns [15] and protecting the temperature of the newborn reduces neonatal morbidity and mortality. No study examining knowledge about newborn bathing has been found; we consider our study to be the first. We determined approximately three-quarters of the healthcare professional participating in our study to be sufficiently knowledgeable about newborn bathing. However, we found that knowledge about newborn bathing was affected by gender, professional group, education level, unit, and institutional setting. In the study, there were more female participants (77.7%) and there was a statistically significant difference (*p* < 0.001) between gender and knowledge levels. It was thought that the reason for this may be the experience of taking care of a child. In addition, when it is considered that female health professionals mostly provide newborn bathing training to mothers, it is thought that the fact that those who give and receive training are also women may create a comfortable and sincere environment in mutual communication and interaction, which may increase the desire to have knowledge. Similarly, it was found that the majority of the health professionals were midwives and that the profession and education level affected the knowledge score. It is thought that this situation is caused by the fact that the education of health professionals in the country where the participants are located is at least at the undergraduate level and only women study midwifery. Likewise, working time also has an effect on this. We can conclude that the repeated use of the basic knowledge acquired through education in working life causes the knowledge to be kept alive and up-to-date. Based on this, it can be said that health professionals working in the field of pediatrics have knowledge about newborn care, including newborn bathing, and this knowledge stems from both their individual characteristics and the pediatric unit they work in. Especially, when it is determined that the majority of them work in the NICU, it may be an indication that neonatal care there is frequently provided by midwives and nurses.

Healthcare professionals should include in their knowledge of newborn bathing, inappropriate bathing practices, their risks, and the complications they can cause [23]. In particular, they are particularly recommended to protect the preservation of skin integrity and to be aware of serious conditions such as heat imbalance. Hypothermia is one of the most common problems encountered in bathing newborns. The Newborn Skin Care Evidence-Based Clinical Practice Guideline, developed by the Association of Women's Health, Obstetric, and Neonatal Nurses (AWHONN), recommends performing the first bath after thermal and cardiorespiratory stability has been achieved and between 6 and 24 h after birth [10]. It has been reported that delaying the first bath of the newborn until 24 h after birth will reduce hypothermia and severe crying for the baby, benefit from the varnish caseosa on the skin, and provide sufficient skin-to-skin contact and mother's participation in the child's bath [11]. In a study provided clinical practice guidance on evidence-based newborn skin care practices from birth to discharge in the hospital environment and concluded that in term newborns, the first bath should be given after thermal and cardiorespiratory stability is achieved, provided the temperature of the environment and bath water is carefully monitored; and recommends giving it approximately 6 h later [15]; previous research shows that bathing healthy newborns at different time points (from one to 9 hours after birth) has no significant effect on infant axillary temperatures [24]. Approximately a quarter of our participants in our study stated that the newborn's first bath should be 24 h after birth, and approximately a fifth stated that it should be done immediately after birth; almost all stated that the newborn should not be given a bath to warm it up. In a region of Ethiopia, the knowledge and practices of midwives and nurses regarding basic newborn care were investigated and found that 183 (67.3%) participants believed that the newborn should be bathed 24 h after birth, and the remaining 89 (32.7%) participants did not know the recommended time for bathing [25]. Another study conducted with 35 nurses to evaluate nurses' knowledge of basic newborn care reported that 86.42% of 21 nurses preferred to bathe the newborn at least 24 h after birth, preferably the second day after birth [26]. First bath time can affect various aspects of newborn care [27]; taking the first bath early may affect the newborn's temperature, blood sugar, bond with the mother, comfort, and safety. In a study, 75% of health professionals reported that bathing should not be done after birth due to hypothermia and 52.9% reported that bathing could be done after 24 h [23]. However, conditions may require bathing earlier, such as heavy meconium staining, excess blood, or chorioamnionitis. It is predicted that delayed newborn bathing will cause more intense skin colonization of resident bacteria. However, it is unclear whether this means an increased risk of early- or late-onset sepsis [28]. The American Association of Pediatrics (AAP) recommends that newborns exposed to human immunodeficiency virus (HIV) through contact with HIV-positive mothers be washed and cleared of secretions as soon as possible after birth [29]; there is no mention of bathing the newborn to prevent vertical transmission of other pathogens [27]. For many parents, it is important to remove visible debris from their babies after the birth process. However, it is suggested that delaying the first bath for both vaginal and cesarean birth is one of the factors affecting mother–baby bonding and early breastfeeding initiation [2, 30]. Our study's results resemble the results of other studies in the literature. Ideally, the timing of the first bath should be determined by the condition of the newborn and the impact of skin-to-skin contact time, breastfeeding initiation, and early mother/family bonding [10, 20, 30–32].

There are structural differences between baby skin and adult skin. In babies, the epidermis is 20% thinner and the stratum corneum is 30% thinner, which increases sensitivity to permeability and dryness [33]. Transepidermal fluid loss increases and stratum corneum hydration decreases in healthy newborns undergoing sponge or wiping bath [34]. In our study, most participants stated that the newborn should be given a wiping/sponging bath. The effect of first bath in the form of wiping/sponging only or bathing with water immediately after birth demands further study.

The United Kingdom National Health Service (NHS) recommends not adding any liquid cleansers to bath water [35]. In the literature, liquid cleansers with a neutral or slightly acidic pH, appropriately formulated for infants, are reported to be healthy and do not affect the maturation of skin barrier function [16]. Almost all our participants stated that the newborn's bath should be done with water only. As a result of a global study conducted with 61 midwives and nurses from 46 countries in Africa, America, Asia, Europe, and Oceania, to determine the national standard care for newborn bathing and the factors affecting it, 43.5% of the participants used soap or cleanser in the newborn bath [36]. And a study examined using water alone and with a skin cleansing product in a newborn's first bath to assess the effect on skin barrier function. One study examined the effect of washing with water only and washing with a skin cleansing product on the skin barrier and reported no difference between their effects compared to each other [2]. Washing newborns with soap and/or similar products after birth affects the integrity of the skin barrier or microbiome [37]. Inappropriate skin cleansing practices may also have a detrimental effect on the immature neonatal skin microbiome, which is an important factor in skin health [38].

The importance of evidence-based practices and their use in the profession should be taught and adopted in the basic education of health professionals [23]. Following this, education on specific topics in primary protection and care practices can be deepened during their specialty education. Within the scope of evidence-based practices and guidelines, training should be provided in primary protection by knowing the ideal bathing method, type, time, duration and materials, and possible risks. Possible risks can be reduced and eliminated with parent education provided with this equipment. In addition, the individual characteristics of parents and babies should also be considered; that is, having skin problems, having a low gestational age, or the characteristics of the environment (altitude, village, town, etc.) can shape parental education. In order for the training to be effective, it should be determined in which area it will be easier for parents to learn (visual, auditory materials, practical expression, etc.). Thus, health professionals obtain care protocols including bathing by following basic education and up-to-date knowledge. Then, if they transfer this information by individualizing it specific to the baby and his/her family, unwanted bathing practices can be prevented and contribute to the adaptation of the baby to the outside world.

## 5. Conclusion

This study revealed health professionals' knowledge of newborn bathing and related issues. Although health sciences education provides instruction about newborn care, only three-quarters of participants had sufficient knowledge. We recommend healthcare education addressing newborn care be improved. We also advocate newborn bathing recommendations be a part of the newborn discharge checklist.

### 5.1. Limitations of the Study

The limitations of the study are that the study is cross-sectional and the population is very large. In addition, the lack of a ready-made measurement tool that measures knowledge and attitude about newborn bathing is another limitation of the study. The strength of the study is the development of the measurement tool and the lack of similar studies to be compared.

### 5.2. Implications for Nursing Management

The impact of the health worker, the patient, and the health system on health outcomes is an integral, contributing factor of healthcare work and crucial for appropriate policy planning. Health systems vary for different health workers and patients. Likewise, the training of health workers should differ according to the age and health needs of the patient. Once national, evidence-based, neonatal care protocols, and standards have been developed, the health system should both support and require health workers to adhere to the standards. The knowledge and practices of healthcare professionals, especially nurses who spend the most active time in neonatal care, play an important role in the safety of newborns. As delayed neonatal bathing has been recommended, it has become critical that all healthcare workers are able to adopt new practices in the workplace. There are only a few studies evaluating the attitudes and care of healthcare workers toward neonatal bathing practices. In light of this, it is thought that this study may attract the attention of healthcare professionals, hospital administrators, and policymakers due to the scarcity of research on this topic. Since the hygiene of newborns directly affects their health, it is necessary to learn the level of knowledge in this field and to develop trainings for this purpose. It is important to update information by considering the factors affecting education. The aim of this study was to determine the determinants of knowledge and attitudes of healthcare workers regarding newborn bathing. Healthcare workers (especially nurses) had sufficient knowledge about neonatal bathing due to their experience and education. It is important to determine the cues for the newborn's first bath. We recommend that studies should be conducted on how healthcare workers convey their knowledge and how mothers apply this knowledge. This is expected to provide an empirical basis for developing an innovation in the management of newborns in order to have an impact on improving health outcomes, both clinically and in the field, especially in healthcare institutions.

## Figures and Tables

**Table 1:** Distribution of participants' knowledge and attitudes about newborn bathing.

Form items		True		False	
		*n*	%	*n*	%
Attitudes	1. It is preferable to wipe the baby with a towel immediately after birth, but if contamination is present, it can be wiped with water	122	87.1	18	12.9
	2. The timing of the first bath should be appropriate to the local culture	36	25.7	104	74.3
	3. Body temperature should be stabilized before the baby's first bath	138	98.6	2	1.4
	4. Health workers should use gloves for the first bath	104	74.3	36	25.7
	6. Routine baths can start before the umbilical cord falls off, but there may be advantages to waiting	130	92.9	10	7.1
	16. When using oils, a mat should be placed in the bath/tub, which should be disinfected regularly	102	72.9	38	27.1

Knowledge	5. The bath will not harm the baby	114	81.4	26	18.6
	7. Bathing is better than washing with a cloth	106	75.7	34	24.3
	8. An evening bath can help calm the baby and improve sleep	138	98.6	2	1.4
	9. Bathing for newborns should last 5–10 min	128	91.4	12	8.6
	10. Newborn bathing should be done 2-3 times a week at a rate appropriate to the local culture	72	51.4	68	48.6
	11. Bathing should take place in a safe place	140	100	0	0
	12. Bathing materials and bath toys should be disinfected to prevent microbiological contamination	140	100	0	0
	13. The water temperature should be 37°C–37.5°C	112	80	28	20
	14. The water depth should be up to the baby's hips	128	91.4	12	8.6
	15. The room air temperature should be 21°C–22°C	90	64.3	50	35.7
	17. Changes in skin texture (e.g., dryness, damage) should be treated with an emollient or protective ointment (diaper area)	140	100	0	0
	18. Bathing provides tactile stimulation and bonding with the baby's parents and other caregivers, making it a fun experience	140	100	0	0
	19. Bathing can be a calming and soothing experience for the newborn	140	100	0	0
	20. The condition of the skin can be affected by environmental factors such as humidity and temperature	140	100	0	0
	21. Softeners should be used when there are signs of dryness, redness, irritation, or any other change in skin texture	132	94.3	8	5.7

**Table 2:** Demographic characteristics of participants (*N* = 140).

**Variables**		**Mean ± Sd**	**Min-max (med)**

Age		31.4 ± 5.9	23–54 (31)
Your professional working time		6.7 ± 4.8	1–15 (6)
Your working time in the last unit you worked in		3.4 ± 2.9	1–15 (2)
Child number		1.2 ± 0.7	1–3(1)

		***n***	**%**

Gender	Female	108	77.7
	Male	32	22.9

Marital status	Married	64	45.7
	Single	76	54.3

Vocation	Midwife	67	47.9
	Physician	36	25.7
	Nurse	37	26.4

Your educational status	Doctorate	3	2.1
	Bachelor's degree	87	62.1
	Specialization in medicine	25	17.9
	Minor specialization	11	7.9
	Master's degree	14	10.0

The unit you are working in	General pediatrics	20	14.3
	Emergency service	17	12.1
	Newborn intensive care unit	49	35
	Baby room	6	4.3
	Gynecology and obstetrics service	12	8.6
	Delivery room	36	25.7

The institution you are working for	Hospital (public)	50	35.7
	Hospital (private)	22	15.7
	Training, research, application hospital	32	22.9
	University hospital	36	25.7

Total		140	100

*Note:* med: median; min: minimum; max: maximum; *n*: number; %: percentage; Sd: standard derivation.

**Table 3:** Participant knowledge and attitudes regarding newborn bathing.

Variables		*n*	%
When do you think a newborn should be given a bathing in bathtub for the first time?	1 week later	16	11.4
	24 h later	36	25.7
	3 days later	8	5.7
	1 week after birth	16	11.4
	Dropping the umbilical cord	32	22.9
	Immediately	26	18.6
	After discharge	6	4.3

How should a newborn's first bath be done?	Bath in a bathtub	28	20
	Wiping/sponging bath	112	80

What kind of cleanser should be used in a newborn's bath?	Antibacterial soap/shampoo	18	12.9
	Solid soap	10	7.1
	Only water	96	68.6
	Liquid soap/shampoo	16	11.4

Should a newborn with low body temperature be given a bath to warm up?	Yes	14	10
	No	126	90

Are there any clues to show that your newborn is ready for a bath?	Yes	98	70
	No	42	30

What do you think are the clues that indicate a newborn is ready for a bath?	No	42	25
	Stable vital signs	12	7.1
	Breastfeeding	2	1.2
	The baby is dirty, oily	6	3.6
	Changes in the skin	12	7.1
	Gas problem	2	1.2
	Irritability, insomnia	4	2.4
	In good health in general	10	6
	Dropping the umbilical cord	20	11.9
	Meconium/bloody	4	2.4
	Absorption of vernix caseosa	38	22.6
	Stability of body temperature	16	9.5

*Note: n*: number; %: percentage.

**Table 4:** Demographic characteristics and information form on newborn bathing level.

**Baby bath knowledge and attitudes status**			***n***	**%**

Total points obtained	Inadequate (16 < pts)		36	25.7
	Adequate (16 ≥ points)		104	74.3

		**IFNB total score**		
**Variables**		**Inadequate (16 <)**	**Adequate (16 ≥)**	**Test value** **p**
		***n***	***n***	

Age				−1.722; 0.085
Child number				**7.585; 0.042**

Gender	Women^a^	36	72	**−3.776** **0.000** ^**∗**^ **a** **>** **b**
	Men^b^	0	32	

Marital status	Married	18	46	−0.597; 0.551^∗^
	Single	18	58	

Your occupation	Midwife^a^	25	42	**9.080** **0.011** ^**∗∗**^ **a** **>** **b; a** **>** **c; b = c**
	Physician^b^	6	30	
	Nurse^c^	5	32	

The unit you are working in	General pediatrics^a^	8	12	**18.637 0.001** ^**∗∗**^ **b > e; c > e; f > e**
	Emergency service^b^	0	17	
	NICU^c^	10	39	
	Baby room^d^	2	4	
	Gynecology and obstetrics^e^	8	4	
	Delivery room^f^	8	28	

Your educational status	Doctorate^a^	0	3	**11.324** **0.023** ^**∗∗**^ **b** **>** **a; b > c; d = e**
	Bachelor's degree^b^	30	57	
	Specialization in medicine^c^	5	20	
	Minor specialization^d^	1	10	
	Master's degree^e^	0	14	

The institution you are working for	Hospital (public)^a^	4	46	**19.227** **0.000** ^**∗∗**^ **a** **>** **d; b = c**
	Hospital (private)^b^	6	16	
	Training, research, application hospital^c^	8	24	
	University hospital^d^	18	18	

*Note:* %: percentage; *n*: number; post hoc tests: Bonferroni, Tamhane; *p* < 0.05; the bold values indicate that the significance is due to midwifery (group a), working in NICU (group b), bachelor's degree (group b) and working in public hospital (group a). The superscript letters are indicated next to each of the categorical data in that row.

Abbreviations: IFNB, information form on newborn bathing; NICU: neonatal intensive care unit

^∗^Mann–Whitney *U*-test.

^∗∗^Kruskal–Wallis test.

## Data Availability

Data are available on request from authors.
